# National survey on internal quality control for tumour markers in clinical laboratories in China

**DOI:** 10.11613/BM.2018.020702

**Published:** 2018-04-15

**Authors:** Wei Wang, Kun Zhong, Shuai Yuan, Falin He, Yuxuan Du, Zhehui Hu, Zhiguo Wang

**Affiliations:** 1 National Center for Clinical Laboratories, Beijing Hospital, National Center of Gerontology, Beijing, P. R. China; 2 Party Committee Office, Beijing Hospital, National Center of Gerontology, Beijing, P.R. China

**Keywords:** internal quality control, tumour marker, imprecision, biological variation

## Abstract

**Introduction:**

This survey was initiated to obtain knowledge on the current situation of internal quality control (IQC) practice for tumour markers (TMs) in China. Additionally, we tried to acquire the most appropriate quality specifications.

**Materials and methods:**

This survey was a current status survey. The IQC information had been collected *via* online questionnaires. All of 1821 clinical laboratories which participated in the 2016 TMs external quality assessment (EQA) programme had been enrolled. The imprecision evaluation criteria were the minimal, desirable, and optimal allowable imprecisions based on biological variations, and 1/3 total allowable error (TEa) and 1/4 TEa.

**Results:**

A total of 1628 laboratories answered the questionnaires (89%). The coefficients of variation (CVs) of the IQC of participant laboratories varied greatly from 1% (5^th^ percentile) to 13% (95^th^ percentile). More than 82% (82 - 91%) of participant laboratories two types of CVs met 1/3 TEa except for CA 19-9. The percentiles of current CVs were smaller than cumulative CVs. A number of 1240 laboratories (76%) reported their principles and systems used. The electrochemiluminescence was the most used principle (45%) and had the smallest CVs.

**Conclusions:**

The performance of laboratories for TMs IQC has yet to be improved. On the basis of the obtained results, 1/3 TEa would be realistic and attainable quality specification for TMs IQC for clinical laboratories in China.

## Introduction

Although the diagnosis of cancer is mostly confirmed by biopsy which has been considered as “gold standard” for a long time, tumour markers (TMs) have an important role in staging and treatment of the cancer (*1*). Internal quality control (IQC) plays a significant role in the routine practice of clinical laboratories. The central role of IQC is to detect clinically important errors and evaluate repeatability in the analytical process. Only when the imprecisions of the measurement system in the laboratory are small enough, the staff might have the opportunity to get satisfactory and reliable results. Clinical laboratories evaluate the imprecisions of their own measurement systems by monitoring monthly (current) and long-time (cumulative) coefficients of variation (CVs) of IQC data, and the CVs are compared with different quality specifications (allowable imprecision criteria). The results of comparison could give the laboratories directions and suggestions to make their performances better. There are several standards which could be used to evaluate the CVs of IQC, such as the specifications based on biological variations including the minimal, desirable, and optimal allowable imprecisions, and 1/3 total allowable error (TEa) and 1/4 TEa. As the evaluation standards of the external quality assessment (EQA) in China were often set as TEa, the 1/3 TEa and 1/4 TEa could be calculated easily for each analyte whose TEa has been known before. Clinical laboratories in China have always used 1/3 TEa and 1/4 TEa to evaluate the imprecisions of their measurement systems (*2*). Although the 1/3 TEa and 1/4 TEa were convenient and easy to use, consideration of combining allowable imprecision specifications based on biological variations and different clinical requirements might be more suitable for all kinds of clinical laboratories regardless of size and conditions, which had gained a consensus recommendation among experts and clinical laboratories staff in recent years (*3*). From each TMs EQA program participating laboratory in China two types of CVs, the control rules, methods, instruments, reagents, calibrators and averages of 6 TMs (alpha-fetoprotein (AFP), carcinoembryonic antigen (CEA), total prostate specific antigen (PSA), cancer antigen 125 (CA125), cancer antigen 15-3 (CA15-3) and cancer antigen 19-9 (CA19-9)) IQC materials were collected. Then all the acquired CVs of IQC were analysed *per* 5 imprecision specifications (minimal, desirable, and optimal allowable imprecision based on biological variations, and 1/3 TEa and 1/4 TEa). After that we could get an overview of measurement imprecision for these 6 TMs in clinical laboratories in China. So far, IQC was one of the best ways to evaluate the imprecision of routine laboratory work in simple, convenient and effective way. There are some different measurement systems used to test TMs in China. We wanted to evaluate and compare their performances, and gain insight on which one is used the most and which one had the smallest imprecision.

## Materials and methods

### Materials

Clinical laboratories participating in the 2016 TMs EQA programme, organized by the National Center for Clinical Laboratories (NCCL) which had been the official EQA programs provider in China for more than 20 years, received survey questionnaires. A total of 1821 laboratories received the questionnaire and 1628 answered it (89%). The TMs included were: AFP, CEA, PSA, CA125, CA15-3 and CA19-9.

### Methods

Our study was a current status survey. In June 2016, all the information was collected *via* a website developed by NCCL for conducting similar studies. The software (Clinet-EQA evaluation system) and website (http://www.clinet.com.cn/) were created by NCCL in 1998. In the beginning, they were used to collect and evaluate EQA program results. In 2016, all the participant laboratories which attended 2016 TMs EQA program were asked to return the investigation results online by the website including the following information: performing IQC or not, principle (the method for concentration determination of analyte), instrument, reagent (manufacturer and batch number), calibrator, number of IQC levels, average of each control (provided by laboratory staff), current CV (May 2016), and cumulative CV of results in-control (the results in-control meaning the results of IQC were in the acceptable ranges set by participant laboratories according to control rules). The CV was calculated and submitted by the participants. We collected two types of CVs: one was the CV of test results in-control for May 2016, the other one was cumulative CV of data in-control (the lot of IQC material unchanged). For the first one, laboratories needed to collect all the results of IQC in May 2016 which were in-control judged by their own control rules, which meant they must remove all the results out-of-control (the results out-of-control meaning the results of IQC were not in the acceptable ranges set by participant laboratories according to control rules) before calculation. For the second one, laboratories had to collect all the results in-control from the first to the last day until May 2016 with the lot of control unchanged. Then, the percentages of IQC CVs of the participants meeting the quality requirements for each TM were calculated *per* 5 imprecision criteria, which were the specifications based on biological variations including the minimal, desirable, and optimal allowable imprecision, 1/3 TEa and 1/4 TEa, respectively.

### Imprecision criteria

Based on the performance evaluation requirements provided by Clinical Laboratory Improvement Amendment (CLIA’ 88), our institute set these criteria as the TEa for these 6 TMs. The 1/3 TEa and 1/4 TEa could be calculated and used to evaluate the imprecisions of the IQC of the participant laboratories in China. There were three imprecision levels which were calculated from biological variations and used to evaluate the performance of IQC, including: 1) minimum performance defined by CV_A_ < 0.75C V_I_ (CV_A_ is analytical precision and CV_I_ is the within-subject biological variation); 2) desirable performance defined by CV_A_ < 0.50 CV_I_; 3) optimum performance defined by CV_A_ < 0.25 CV_I_. The data we used referred to the biologic variation list provided by Ricos *et al.* (*4*).

### Statistical analysis

Data were analysed using SPSS 13.0 (SPSS Inc, Chicago, IL, USA) and Clinet-EQA evaluation system V1.0 (Clinet Corp, Beijing, China), designed by NCCL and used in the national EQA program (see http://www.clinet.com.cn/shop/shop). The distributions of CVs of each analyte were tested by Kolmogorov-Smirnov test for normality. The related statistical parameters of CVs, including median, the 5th, 25th, 75th, and 95th percentile were calculated. The percentages of laboratories (total and divided into different subgroups by measurement systems) that met quality requirements of imprecision were calculated.

## Results

The questionnaire was answered by 1628 laboratories (1628/1821, 89%). Laboratories which replied the survey submitted the related information and data for more than one TM. Table 1 shows the definitions of survey items. Table 2 shows the quality specifications of TMs based on CLIA’88 and biological variations, while Table 3 shows the numbers of participant laboratories, medians, other percentiles of two types of CVs, and the percentages of laboratories meeting quality requirements. Table 4 shows the principles and systems used for testing TMs; Table 5 and 6 show the percentages of two types of CVs of different principles and systems meeting different imprecision criteria. These two types of CVs were shown abnormal distributions accessed by normality test.

**Table 1:** The content of the tumour marker IQC survey

**Item**	**Definition**
Performing TMs IQC or not?	If your laboratory have performed TMs IQC, choose yes otherwise choose no
Principle	The method of determination of concentration of analyte
Instrument	Manufacturer and model
Reagent	Manufacturer and lot
Calibrator	Manufacturer and lot
Number of IQC controls	How many levels
Average of each control	The average in the TMs IQC chart
Current CV	The CV of all TMs IQC results in-control
Long-time (cumulative) CV of results in-control	The CV of all TMs IQC results in-control from the first day to the last until May 2016 with the lot unchanged
TMs - tumour markers. IQC - internal quality control. CV - coefficient of variation.	

**Table 2:** Quality specifications of tumour markers based on CLIA’88 and biological variation

**Analyte**	**1/3 TEa (%)**	**1/4 Tea (%)**	Minimum performance (%)*	Desirable performance (%)*	Optimum performance (%)*
AFP	8	6	9	6	3
CEA	8	6	10	6	3
PSA	8	6	14	9	5
CA125	8	6	19	12	6
CA15-3	8	6	5	3	2
CA19-9	8	6	12	8	4
TMs - tumour markers. TEa - total allowable error. AFP - alpha-fetoprotein. CEA - carcinoembryonic antigen. PSA - total prostate specific antigen. CA125 - cancer antigen 125. CA15-3 - cancer antigen 15-3. CA19-9 – cancer antigen 19-9. *Performance according to biological variation.					

**Table 3:** Survey results for the participating laboratories

**Type of CV**	**TM**	**N**	**CVs (%)**					**Laboratories meeting allowable imprecision specifications based on CLIA’88 and biological variation (%)**				
**Median**	**5th**	**25th**	**75th**	**95th**	**1/3 TEa**	**1/4 TEa**	**Minimum**	**Desirable**	**Optimal**
**Current**	AFP	1358	5	2	3	7	10	88	69	91	67	24
	CEA	1367	5	2	3	7	10	88	70	92	70	24
	PSA	1192	5	1	3	7	11	87	69	98	91	46
	CA125	1263	4	1	3	6	10	91	75	100	98	74
	CA15-3	1199	5	2	3	7	11	89	69	45	20	3
	CA19-9	1247	6	2	4	8	12	78	58	95	75	29
**Cumulative**	AFP	1243	5	2	4	7	11	84	62	88	58	14
	CEA	1248	5	2	4	7	12	82	61	89	62	14
	PSA	1098	6	2	4	7	12	83	61	97	88	35
	CA125	1155	5	2	4	7	10	88	65	99	97	64
	CA15-3	1102	6	2	4	7	12	84	59	33	10	2
	CA19-9	1146	6	2	4	9	13	73	50	92	69	20
CVs - coefficient of variations reported by participating laboratories. TM - tumour marker. TEa - total allowable error. N – number of participating laboratories. 5th, 25th, 75th, 95th – percentiles. AFP - alpha-fetoprotein. CEA - carcinoembryonic antigen. PSA - total prostate specific antigen. CA125 - cancer antigen 125. CA15-3 - cancer antigen 15-3. CA19-9 – cancer antigen 19-9.												

**Table 4:** Principles and systems for tumour markers assays

**Group**	**Assay principle**	**System (manufacturer, city, country)**	**Participating laboratories, N**					
**AFP**	**CEA**	**PSA**	**CA125**	**CA15-3**	**CA19-9**
1	Acridine ester direct chemiluminescence	Abbott Architect i2000SR/i2000/i1000SR (Abbott,Chicago, USA)	224	223	213	203	197	183
		Siemens Centaur /XP (Siemens, Berlin, Germany)	122	125	73	113	106	112
		Siemens Centaur CP (Siemens, Berlin, Germany)	22	22	15	18	16	18
2	Microparticle chemiluminescence - AMPPD labelling	Beckman Access/Access 2 (Beckman, Brea, USA)	27	27	25	26	24	23
		Beckman DXI 600, DXI 800 (Beckman, Brea, USA)	139	138	117	117	114	119
		Shenzhen Mindray CL-2000i (Shenzhen, Guangdong, China)	26	26	24	24	22	23
3	Luminol/Isoluminol chemiluminescence	DiaSorin S.p.A LIAISON (DiaSorin, Saluggia, Italia)	17	19	15	15	15	16
		Maglumi 600/1000/1000plus/2000/2000plus/3000/4000 (Shenzhen, Guangdong, China)	35	36	32	34	33	35
4	Flow fluorescence immunoassay	Luminex100, 200 (Luminex, Austin, USA)	30	31	28	31	27	29
5	ECL	Roche Cobas e411 (Roche, Basel, Switzerland)	84	85	79	84	81	85
		Roche Cobas e601/e602 (Roche, Basel, Switzerland)	410	415	383	404	384	406
		Roche Modular E170/PP/P800 (Roche, Basel, Switzerland)	60	61	55	58	57	61
6	Enzyme immuno- chemiluminescence	AutoLumo A2000 (Zhengzhou, Henan, China)	31	32	25	27	26	26
TMs - tumour markers. ECL – electrochemiluminescence. AFP - alpha-fetoprotein. CEA - carcinoembryonic antigen. PSA - total prostate specific antigen. CA125 - cancer antigen 125. CA15-3 - cancer antigen 15-3. CA19-9 – cancer antigen 19-9. AMPPD - 3-(2’-spiroadamantane)-4-methoxy-4-(3”-phosphoryloxy)-phenyl-1,2-dioxetanes.								

**Table 5:** Proportions of laboratories meeting different imprecision specifications with current coefficients of variation

**TM**	**Principle of assay (group)**	**Allowable imprecision specifications based on CLIA’88 and biological variation (%)**				
**1/3 TEa**	**1/4 TEa**	**Minimum**	**Desirable**	**Optimal**
**AFP**	1	86	72	70	89	29
	2	86	64	63	90	15
	3	79	63	62	83	12
	4	77	27	23	87	3
	5	95	76	73	97	28
	6	52	32	29	65	6
**CEA**	1	84	67	67	89	14
	2	89	73	74	92	19
	3	73	49	49	82	5
	4	87	42	42	97	6
	5	95	79	79	97	37
	6	56	41	41	72	13
**PSA**	1	84	69	86	98	41
	2	87	73	91	98	50
	3	79	51	89	98	19
	4	86	50	96	100	4
	5	94	74	97	99	57
	6	56	32	64	92	12
**CA125**	1	92	83	98	100	82
	2	89	79	98	100	78
	3	82	59	98	100	59
	4	74	23	94	100	23
	5	96	79	100	100	78
	6	59	37	78	100	37
**CA15-3**	1	87	69	14	45	3
	2	89	69	11	37	3
	3	75	44	8	27	6
	4	78	33	0	7	0
	5	95	76	29	56	3
	6	54	23	8	12	8
**CA19-9**	1	65	36	59	91	9
	2	88	72	85	95	25
	3	71	43	65	96	27
	4	69	17	62	97	3
	5	88	74	86	99	45
	6	27	23	27	77	12
TM - tumour marker. Group 1 – acridine-ester direct chemiluminescence. Group 2 - microparticle chemiluminescence. Group 3 - luminol/isoluminol chemiluminescence. Group 4 - flow fluorescence immunoassay. Group 5 – electrochemiluminescence. Group 6 - enzyme immunochemical luminescence. TEa - total allowable error. AFP - alpha-fetoprotein. CEA - carcinoembryonic antigen. PSA - total prostate specific antigen. CA125 - cancer antigen 125. CA15-3 - cancer antigen 15-3. CA19-9 – cancer antigen 19-9.						

**Table 6:** Proportions of laboratories meeting different imprecision specifications with cumulative coefficients of variation

**TM**	**Principle of assay (group)**	**Allowable imprecision specifications based on CLIA’88 and biological variation (%)**				
**1/3 TEa**	**1/4 TEa**	**Minimum**	**Desirable**	**Optimal**
**AFP**	1	82	64	60	85	19
	2	77	57	49	84	7
	3	72	46	41	76	9
	4	73	20	17	90	0
	5	91	69	67	95	16
	6	52	30	30	63	7
**CEA**	1	74	54	55	83	6
	2	84	60	62	88	8
	3	57	37	37	69	12
	4	84	26	29	94	3
	5	92	72	73	96	24
	6	56	30	30	78	11
**PSA**	1	79	57	84	97	27
	2	80	61	85	97	31
	3	72	49	81	98	30
	4	86	29	93	96	4
	5	91	68	93	97	46
	6	59	36	64	95	14
**CA125**	1	86	63	97	99	63
	2	85	67	96	99	63
	3	73	40	98	100	40
	4	74	16	94	97	16
	5	94	74	99	100	73
	6	61	35	78	100	35
**CA15-3**	1	78	56	5	29	2
	2	79	52	5	23	1
	3	64	36	9	18	7
	4	96	15	0	0	0
	5	91	69	15	44	2
	6	55	14	9	9	9
**CA19-9**	1	53	26	48	85	7
	2	83	54	78	94	15
	3	64	38	57	85	26
	4	76	14	62	93	3
	5	84	66	82	97	30
	6	32	23	32	77	9
TM - tumour marker. Group 1 – acridine-ester direct chemiluminescence. Group 2 - microparticle chemiluminescence. Group 3 - luminol/isoluminol chemiluminescence. Group 4 - flow fluorescence immunoassay. Group 5 – electrochemiluminescence. Group 6 - enzyme immunochemical luminescence. TEa - total allowable error. AFP - alpha-fetoprotein. CEA - carcinoembryonic antigen. PSA - total prostate specific antigen. CA125 - cancer antigen 125. CA15-3 - cancer antigen 15-3. CA19-9 – cancer antigen 19-9.						

In Table 3 current CVs are listed and 5 out of 6 analytes (except CA19-9) showed relatively smaller CVs compared with CA19-9. The percentages of laboratories whose CVs were smaller than 1/3 TEa specification for these TMs were all above 87% (from 87% for PSA to 91% for CA125), while the percentages varied markedly (from 3% for CA15-3 to 74% for CA125) when the optimal allowable imprecision specification was used. Although current CVs of CA19-9 were larger than other TMs, the percentage of laboratories whose CVs were smaller than 1/3 TEa specification was close to 80% (974/1247). The percentages of laboratories whose CVs met imprecision criteria based on biological variations for CA15-3 were significantly smaller than other TMs. For the cumulative CVs, as the current ones, 5 TMs got relatively smaller CVs compared with CA19-9. The percentiles of current CVs of laboratories were lower than cumulative CVs.

A proportion of 76% laboratories (1240/1628) reported their principles and systems used. Table 4 shows the principles, systems, and numbers of laboratories of each system as well as the manufacturer’s information. We categorized the principles of assay into 6 groups including: 1) acridine ester direct chemiluminescence; 2) microparticle chemiluminescence-3-(2’-spiroadamantane)-4-methoxy-4-(3”-phosphoryloxy)-phenyl-1,2-dioxetanes (AMPPD) labelling; 3) luminol/isoluminol chemiluminescence; 4) flow fluorescence immunoassay; 5) electrochemiluminescence (ECL); 6) enzyme immunochemical luminescence. The ECL was the most used of all the 6 principles. About 45% of laboratories used ECL principle (Group 5) to test TMs. The principles used fewest were flow fluorescence immunoassay (Group 4) and enzyme immunochemical luminescence (Group 6), the percentages were all about 3%. Coincidentally, the percentages of group 5 meeting the specifications were the highest while group 4 and 6 were the lowest.

Table 5 and table 6 show the information about the percentages of current CVs and cumulative CVs meeting different imprecision criteria for all groups. The results of group 5 were better compared to others. Almost all the assay principles could archieve more than 80% of laboratories meeting the minimum imprecision criterion based on biological variation except CA15-3. However, judging by the strictest specification based on biological variations (*i.e.* the optimal), almost all the percentages were lower than 50%. The results of group 6 were not so encouraging for almost all the imprecision criteria.

## Discussion

The results of our survey suggest that there were remarkable variations for the TMs IQC in China, including manufacturer, principle and imprecision. Harmonized control rules with defined ranges of imprecisions were not defined in China. Some studies only kept their focus on the performances of different test systems (*5*). Compared with Bertsch *et al.* the range of CVs for CA19-9 IQC was much wider in our study. The performances should be improved later (*5*). Consequently, the lack of standards might lead to many problems in daily monitoring of imprecision. The wide ranges of current and cumulative CVs of TM IQC materials reported by the participant laboratories, which varied dramatically (from less than 1% to more than 50%) also shocked us.

Cancer antigen 19-9 had the largest CVs of all TMs, which presented that Chinese laboratories should pay more attention to this analyte. The cumulative CVs were slightly larger than current CVs, which might reveal that the long-time repeatability was not as good as short-term one. We might provide some suggestions such as: 1) about 80% Chinese clinical laboratories with better medical resource could meet the 1/3 TEa criteria which could be considered as evaluation standard for IQC program; 2) the cumulative CVs were larger than current CVs, which means that more attention should be paid to long-time repeatability. The ideal situation should be, the longer a lot of IQC material tested, the smaller CVs should be. That was because with the increasing of test times, the results and variation should be more and more stable, if the test system had been stable all the time. The long-time stability of measurement system should be improved in China.

Cancer antigen 15-3 had the strictest specification based on biological variation, which made the percentages of laboratories meeting criteria much lower than other TMs. So we should know that 1/3 TEa should be used carefully because it was much bigger than the specifications based on biological variation. We might pay more effort on finding a better and more reasonable IQC evaluation criterion which could better match the performance of test system and biological variations in the future.

The ECL method had the highest CVs of TMs IQC meeting the evaluation specifications, which might be one of the reasons why most laboratories chose ECL method. A study also verified that the ECL method and Roche test system could achieve a good performance in TMs measurement (*6*). Compared with other analytes in Chinese Proficiency testing (PT) panel, creating national evaluation specifications for imprecision of TMs was needed to improve the quality of measurement (*7*, *8*).

Clinical laboratories in this study were located in different areas of China and could be considered as representatives of Chinese clinical laboratories with better medical resources and more concern on their performance than those who did not participate to TMs EQA program or return the questionnaires. Most of the clinical laboratories participating in these kinds of surveys were mostly first-class hospitals in China (*9*, *10*).

There were still some limitations in our study. Human epidermal growth factor receptor 2 (HER2) was a TM which was newer than these 6 TMs and it had been in focus in recent years. There is another way to perform IQC for HER2 and we should keep up with the time (*11*). Another study reported that lyophilized QC materials for TMs were insufficiently stable for use in quality control among clinical laboratories (*12*). This might result in cumulative CVs bigger than current ones, which might be studied in the further studies. In Lent *et al.* study, they made a conclusion that treatment errors in association with PSA determinations could therefore be uniformly and plausibly assessed using objective criteria and could thus be avoided (*13*). IQC was an effective way to decrease errors occurrence. We might combine IQC to evaluate treatment errors occurrence rate to study the relationship between them.

In conclusion, the performance of laboratories for TMs IQC has yet to be improved. On the basis of the obtained results, 1/3 TEa would be realistic and attainable quality specification for TMs IQC for clinical laboratories in China.
